# A Post-operative Masquerade: Simulation-based Scenario Challenging Clinical Clerks to Recognize an Atypical Presentation of Myocardial Infarction

**DOI:** 10.7759/cureus.7510

**Published:** 2020-04-02

**Authors:** Vanessa Pickard, Noel B O'Regan, Gillian Sheppard, Adam Dubrowski

**Affiliations:** 1 Medicine, Memorial University of Newfoundland/Janeway, St. John's, CAN; 2 Anesthesiology, Memorial University of Newfoundland/Janeway, St. John's, CAN; 3 Emergency Medicine, Memorial University of Newfoundland, St. John's, CAN; 4 Health Sciences, Ontario Tech University, Oshawa, CAN

**Keywords:** simulation-based medical education, cardiology, perioperative myocardial infarction, medical response

## Abstract

Post-operative myocardial infarctions (MI) are a challenging diagnosis due to the alterations in the presenting complaint compared to an acute MI. Patients may be asymptomatic due to their anesthetics and sedatives from their operation which may create clinical confusion. As such, there is an increased risk for delayed administration of reperfusion therapies in this patient population which has shown to increase morbidity and mortality. It is anticipated that the difficulty of recognizing a post-operative MI would be exacerbated for clinical clerks due to their lack of clinical experience and overstimulation. Fortunately, the use of simulation-based learning has been proven to be a useful teaching tool to help clinical clerks manage medical problems in a controlled environment. This technical report describes a simulation case designed to enhance the recognition and response to a post-operative MI by a third-year clinical clerk. In this scenario, a 56-year-old male accountant presents with shortness of breath while recovering in the orthopaedic ward 12 hours following a total knee replacement (TKR). The clinical clerks are expected to conduct an independent follow-up prior to finishing their shift during which the patient begins complaining of shortness of breath. The clerk is required to order an electrocardiogram (ECG) for further analysis which reveals an anterior ST-segment elevation. Once recognized, a request for the crash cart and patient handover to the senior physician are expected.

## Introduction

Advancements in medicine have increased the number of patients undergoing non-cardiac surgeries, which are associated with elevated cardiac risk in the perioperative period [1]. It has been shown that patients who experience a myocardial infarction (MI) following a non-cardiac surgery have a 15%-25% increased in-hospital mortality rate [2]. Fortunately, the use of reperfusion therapies has demonstrated improved survival rates when implemented in a timely fashion [3]. For each minute that therapy is delayed, there is a subsequent increase in the probability of mortality and morbidity [4]. 

Time-delay in administering reperfusion therapies is of concern due to the difficulty in recognizing an MI in the perioperative period [5]. Patients often present as asymptomatic due to a combination of anesthetics and sedatives, and the skills required to recognize a perioperative MI differ from those required to recognize an acute MI [5,6]. Furthermore, evidence suggests when novice learners, such as clinical clerks, are immersed in busy environments, their cognitive resources are depleted rapidly which may be linked to an inability to make clinical decisions [7]. It is, therefore, anticipated the difficulty of recognizing a perioperative MI would be exacerbated for clinical clerks due to their lack of experience and overstimulation. This difficulty may increase patient risks through further time delay when a clinical clerk is conducting a follow-up and required to make an independent judgement without the presence of their attending physician. 

Clinical clerks are often the first members to check in with a patient in the post-operative period. However, due to their limited clinical exposure, it may be difficult to recognize a masqueraded MI in the post-operative period which may have serious ramifications for the patient. Simulation-based learning could be beneficial in teaching clinical clerks how to recognize and respond to the signs of a post-operative MI in a controlled clinical setting [8]. Specifically, it has been shown that learners who acquire skills in simulation show reduced stress levels, leading to improved practice and performance [9-11]. Simulation-based learning has been implemented as a tool to teach clinical clerks in the past. For example, Hogg et al. showed improved recognition and response of third-year clinical clerks to clinical deterioration in adult patients after a period of simulation-based practice [12]. 

The purpose of the current technical report is to describe a simulation scenario that can be used to enhance the recognition and response to a post-operative MI by a third-year clinical clerk. The learning objectives of this simulation-based scenario were to enable third-year clinical clerks to: (1) Perform a focused history and physical exam for shortness of breath and diaphoresis, (2) recognize when an electrocardiogram (ECG) is required in the post-operative period, (3) recognize a post-operative MI following a non-cardiac surgery, and (4) activate an appropriate medical response including ordering a crash cart and calling for help from an attending physician. Learners should be able to successfully complete the simulation-based learning scenario and develop the skills and competency necessary to respond to a post-operative MI. Specifically in this simulation scenario, third-year clinical clerks acquire the skills necessary to recognize a post-operative MI following a total knee replacement (TKR) surgery. The patient in the scenario is a 56-year-old male accountant who presents with shortness of breath while recovering in the orthopaedic ward 12 hours following the surgery. The clerks will be asked to conduct an independent patient follow-up prior to completing their shift. During the follow-up assessment, the patient begins complaining of shortness of breath and appears diaphoretic. Next, they will be required to order an ECG for further analysis which reveals an anterior ST-segment elevation. Once recognized, the clerk will request for the crash cart and patient handover to the senior physician must follow immediately. The simulation is designed to take place in a university-based simulation facility; however, it can be modified to other contexts.

## Technical report

Learning objectives

The learning objectives of this simulation-based scenario were to enable third-year clinical clerks to:

1. Perform a focused history and physical exam adapted to the clinical scenario 

2. Recognize when an ECG is required in the post-operative period

3. Recognize an anterior ST-segment elevation in the post-operative period following a non-cardiac surgery

4. Activate an appropriate medical response including ordering a crash cart and performing handover to the attending physician

Case

A 56-year-old male accountant presents with shortness of breath while recovering in the orthopaedic ward 12 hours following a TKR. The patient is responsive, appears diaphoretic but has no complaints of concomitant chest pain. Vital signs show a heart rate (HR) of 100 beat per minute (BPM), blood pressure (BP) of 160/90 and 98% O2 saturation (SaO2) with a respiration rate (RR) of 24 breaths per minute (BrPM). The patient received a spinal anesthetic with an adductor canal nerve block prior to the surgery. The patient is currently on a Patient Controlled Analgesia (PCA) pump which releases 2 mg of morphine on patient demand with a 5-10-minute lockout period with a maximum dosage of 10 mg of morphine per hour. Reviewing the patient’s chart reveals that the patient has a body mass index (BMI) of 34 and is on simvastatin 40mg PO once daily and quinapril 20mg PO once daily. The patient has no known history of allergies. There is a single nurse confederate in the room.

Context 

The simulation was designed to teach third year clinical clerks the skills to recognize a post-operative MI following a TKR and takes place in a university-based simulation facility. Twelve hours following a TKR, the clinical clerk conducts an independent patient follow-up prior to finishing their shift. During the follow-up assessment, the patient begins complaining of shortness of breath and is diaphoretic. The clinical clerk will request supplemental oxygen for the patient and is required to perform a focused history to formulate a prioritized differential diagnosis with cardiac causes as the preferred system. The clinical clerk will then order an ECG for further analysis which reveals an anterior ST-segment elevation. Once recognized, the clerk should request for the crash cart and patient handover to the senior physician must immediately follow for initiation of reperfusion therapy.

Inputs

The personnel required to run this simulation include learners, a standardized patient, and technical staff to instruct the standardized patient. The targeted learners for this scenario are clinical clerks, with a minimum of three months of experience in clinical clerkship. An optimal learner would have clerkship experience specifically in orthopaedics but any surgical field is acceptable. The assessor is the primary individual responsible for the formative assessment of the learner and of the simulation-based scenario. The simulation scenario requires the use of a standardized patient exhibiting signs of shortness of breath and distress in a clinically controlled setting. An ECG displaying an anterior ST-segment elevation is required as a component for the learner to analyze and interpret. Additionally, an advanced cardiovascular life support (ACLS) cart is needed for when the ST-segment elevation is recognized. A nurse confederate is used to provide prompts if there is no action taken by the learner. 

Process 

The learners undergo a pre-briefing session where they are instructed they are practicing a follow-up assessment on a patient 12-hours following a TKR. A fiction contract is signed by the learners which highlights the limitations and fidelity of the simulation scenario [13]. During this time, the *basic assumption* is reviewed which highlights the notion that all learners are regarded as intelligent individuals who will put forth an honest and sincere effort [13]. The purpose of both the fiction contract and basic assumption are to ensure that there is a safe learning environment. Due to the evaluation of a timely response, the learners will not be informed of the underlying goal to be able to recognize a post-operative MI following a non-cardiac surgery. 

Following the pre-briefing session, the learners are instructed to enter into a controlled, clinical simulation setting and are provided with the pre-scenario information before beginning the follow-up assessment (Table 1). During the follow-up, the standardized patient verbalizes they are experiencing shortness of breath but they are not experiencing concomitant chest pain. Absence of angina increases the difficulty in recognizing the MI for the clinical clerk; however, it is expected they order an ECG for further analysis. Following the ECG analysis of the ST-segment elevation, the learner is expected to engage in the necessary medical action by calling for a crash cart and initiating patient handover with the attending physician. 

**Table 1:** Background information and a stepwise scenario template submitted to the simulation technical staff responsible for providing the equipment and preparing the standardized patient

Pre-Scenario			
You are a third-year medical clerk. A 56-year-old accountant presents with shortness of breath while recovering in the orthopaedic ward 12-hours following a total knee replacement (TKR). The patient received a spinal anesthetic with an adductor canal nerve block prior to the surgery. The patient is currently on a Patient Controlled Analgesia (PCA) pump which releases 2 mg of morphine on patient demand with a 5-10 minute lockout period with a maximum dosage is 10 mg of morphine per hour. The medical chart is available for your review and there is a single nurse confederate in the room.			
History			
Allergies	Penicillin, peanuts		
Medications	Simvastatin 40 mg PO once daily, quinapril 20 mg PO once daily, morphine 10mg/hour		
Past Medical History	Hypertension, hypercholesteremia		
Social History	Accountant, married with 3 grown children, smoker, occasional cocaine use		
Family History	Mother died from bowel cancer at age 76, father and two brothers are alive with controlled hypertension, one sister with type 2 diabetes		
Initial Vitals	Heart rate (HR) 84, blood pressure (BP) 110/76, SaO2 92%		
Physical Exam	Appears pale and diaphoretic. Alert and oriented to person, time and place but moderately distressed. Good air entry bilaterally, no adventitia, showing increased WOB. Irregular heart sounds: S1, S2 and S3, JVP 6cm, no murmur or precordial rub. No unilateral leg swelling or calf tenderness.		
Initial Expected Actions			
1. Focused History and Physical Exam			
2. Order ECG			
3. Recognize Anterior ST Segment Elevation			
4. Order Crash Cart			
5. Call for Help and Provide Handover to Attending Physician			
Begin Scenario			
Objective 1: Recognize when an ECG is required in the perioperative period			
Stage	Vitals	Expected Actions	No Action by Learner
Patient complains of shortness of breath and appears diaphoretic	HR 84, BP 110/76, SaO2 92%	Focused cardiac history and physical exam, order ECG	Prompt by confederate nurse: “I used to work in the cardiac care unit and it looks like he is having an infarct”
Objective 2: Recognize a perioperative MI following a non-cardiac surgery			
Stage	Vitals	Expected Actions	No Action by Learner
ECG Analysis	HR 100, BP 90/60, SaO2 92%	Recognize anterior ST-segment elevation	Prompt by confederate nurse: “He doesn’t look very good. I am going to get the crash cart”
Objective 3: Activate an appropriate medical response including ordering a crash cart and calling for help from an attending physician			
Stage	Vitals	Expected Actions	No Action by Learner
Initiate medical response	HR 100, BP 80/50, SaO2 88%, ECG shows Sinus tachycardia and premature ventricular contractions (PVCs) and anterior STEMI	Order crash cart, call for help from senior physician	
Patient handover	HR 100, BP 80/50, SaO2 88%	Provide patient details to senior physician	

Following the simulation scenario, there is a debriefing protocol with the assessor. A systematic review of high-fidelity simulation literature has reported that feedback, including aspects like debriefing, is the most important component of medical-based simulation learning [14]. Once the simulation scenario is complete, the learners are asked to leave the room to receive their feedback. The feedback follows the LEARN framework that has been developed to provide an effective debrief for learners engaged in a simulation-based learning scenario [15]. The first step involves the assessor re-visiting the learning objectives in response to the performance of the learner. Following this, the assessor invites the learners to express their emotions regarding the simulation-based scenario. This is a useful step for learners to express themselves and be able to focus on discussing their learning in the next steps. Additionally, it can assist the assessors in reforming and evaluating the simulation scenario as they can get insight on sources of frustration. The third step of the debrief is designed to focus on actions and reflections through direct feedback as recommended for novice assessors. Direct feedback enables the assessor to close the gap in knowledge and in skill. The last step of the debrief includes the next steps where there is an opportunity for the learner to describe one thing they have learned. The debrief session is followed with an opportunity for the learners the engage in the simulation scenario for a second time to consolidate the information and experience the value of applying new knowledge [15].

Products/outcomes

The performance of the learners is assessed using a combination of assessment techniques. The products are assessed using a procedural specific checklist in conjunction with a Objective Structured Clinical Exam (OSCE) assessment tool adapted to this clinical scenario. The procedural specific checklist includes a step-wise progression of clinical tasks that are required to recognize and respond to a post-operative MI effectively. For this clinical simulation scenario, the clinical tasks include performing a focused history and physical exam, requesting an ECG, recognizing the ST-segment elevation, requesting a crash cart and performing a patient handover to the senior physician on staff. Each of these tasks are evaluated in a dichotomous fashion where a trained clinical expert indicates if the medical clerk was successful by circling “yes” or “no” on the checklist (Table 2). 

**Table 2:** A procedural specific checklist of tasks required by the medical clerk to recognize and respond to a post-operative myocardial infarction (MI) The start and end time of the simulation is recorded along with the time at which each procedural specific task is completed.

Procedural Specific Checklist				
Start Time: End Time:				
Action Item Recognizing and responding to a post-operative myocardial infarction (MI)	Action Completed? (circle one)		Time	
Performs a brief, focused history and physical exam	Yes	No
Orders an ECG	Yes	No
Recognizes the ST-segment elevation	Yes	No
Orders the advanced cardiovascular life support (ACLS) cart	Yes	No
Pages the attending physician and initiates patient handover	Yes	No

In contrast to the step-by-step assessment of the execution of the tasks, the quality of the overall performance of these tasks is assessed using a carefully crafted OSCE. The OSCE is an assessment tool that has been historically used to assess the clinical competence of medical learners under a variety of simulated clinical scenarios. Behaviourally anchored rating scales (BARS) were created for the OSCE to assess the quality of the learner's performance in this clinical simulation based scenario (Table 3).

**Table 3:** A detailed Objective Structured Clinical Exam (OSCE) to evaluate the quality of performance of the learner in recognizing and responding to a post-operative myocardial infarction (MI) on a behaviourally anchored rating scale (BARS) A pass/fail score for an OSCE can be found at the bottom.

Components of the Objective Structured Clinical Exam	Poor	Fair	Good	Excellent
Communication skills and professionalism	Interrupts the patient frequently and appears frantic. The learner demonstrates difficulty in putting the patient at ease.	Communicates their assessment and decision making with the patient and nurse confederate. Appears uncomfortable in the situation but has reasonable control given their level of training.	Appropriate communication with the patient and nurse confederate. Communicates their assessment and decision making and appears to be in control of the situation at a level that would be appropriate given their training.	Appears confident, calm and in control of the situation. Places the patient at ease while effectively gathering information from both the patient and nurse. Communication with the staff and patient is at a level that would be above expectations for their level training.
Focused history	Demonstrates knowledge below their level of training. Fails to perform a focused history to obtain further information about the clinical picture. Does not screen for further risk factors and reacts based on the limited information provided in the prompt.	Demonstrates knowledge below their level of training. Performs a focused history but is limited to only a few screening questions (<3). Misses the majority of risk factors that would lead to cardiac causes (smoking, obesity, diabetes, hyperlipidemia, hypertension, cocaine use, previous coronary artery disease, family history of heart disease).	Demonstrates knowledge appropriate to their level of training. Pertinent questions to the clinical scenario are asked leading to a prioritized differential diagnosis including both cardiac and respiratory causes. The majority of risk factors were screened for except 2-3 (obesity, smoking, coagulopathies, bleeding disorders, malignancy, recent trauma, recent long distance travel, cough, unilateral leg swelling, diabetes, hyperlipidemia, hypertension, cocaine use, previous CA, family history of heart disease).	Demonstrates knowledge beyond their level of training and applies it appropriately to this situation. Asks pertinent questions to formulate a prioritized differential diagnosis ultimately ruling out respiratory causes and narrowing in on cardiac causes. Nearly all relevant risk factors were accounted for except 1 in a timely fashion (obesity, smoking, coagulopathies, bleeding disorders, malignancy, recent trauma, recent long distance travel, cough, unilateral leg swelling, diabetes, hyperlipidemia, hypertension, cocaine use, previous CA, family history of heart disease).
Focused physical exam	Does not perform a physical exam.	Performs a limited physical exam only listening to breath sounds. The learner describes the breath sounds out loud to the assessor.	Performs an appropriate physical exam including respiratory and precordial examinations. The learner describes the patient's breath sounds, adventitia, work of breathing, heart sounds, murmurs and precordial rubs. Vital signs are asked to be repeated.	Performs a physical exam that is complete and timely. Begins with the learner commenting on the patient's appearance (diaphoretic, increased WOB, signs of cyanosis). The learner describes a midline trachea, breath sounds, adventitia, heart sounds, murmurs, precordial rubs and JVP. The learner also assesses for calf tenderness, unilateral leg swelling and asks for vital signs to be repeated.
Initiating response to a perioperative MI	Does not identify the situation as a cardiac cause and must be prompted by the nurse confederate to get an ECG. Fails to recognize the anterior ST-elevation MI.	Identifies respiratory distress and calls for supplemental oxygen and investigations/management for respiratory pathology (i.e. chest X-ray, CT angiogram and/or chest tube). Must be prompted by the nurse confederate to consider cardiac causes and order an ECG. Recognizes the anterior ST-elevation MI and calls the attending physician.	Identifies a cardiac cause for the patient's presentation and promptly orders an ECG. Recognizes an anterior ST-elevation MI and immediately calls the attending physician for further management.	Identifies a cardiac cause for the patient's presentation and promptly orders and ECG and recognizes an anterior ST-elevation MI. The learner orders management steps that would be appropriate given their position (i.e. supplemental oxygen, crash cart, prepares nurses for MI). Calls the attending physician to provide handover and initiate reperfusion therapy.
Patient handover to the attending physician	Learner fails to provide handover to the attending physician. The nurse confederate must prompt the learner to call the attending physician for further assistance.	Learner provides insufficient information to the attending physician for handover leaving many gaps in knowledge. The handover that was provided would require the attending physician to repeat a focused history and physical exam to obtain the appropriate information.	Learner provides most of the pertinent information for a successful handover to the attending physician. The information is presented in a slightly disordered pattern but in a clear and comprehensible manner.	Learner provides all of the pertinent information for a successful handover to the attending physician. The information is provided in a clear and concise manner that flows effortlessly and logically.
Overall, on this task, should this learner: Pass Fail					

## Discussion

Anesthetics administered in the post-operative period will alter the presenting symptoms of an MI. Many patients will not experience any form of chest pain which often serves as the red flag symptom for many clinical clerks. A cohort study conducted by Devereaux et al. in 2011 found that 65.3% of patients did not experience any ischemic symptoms during their perioperative MI [3]. The absence of these symptoms may make the recognition of an MI more difficult for medical trainees. Indeed, this difficulty may be increased due to the inexperience in the postoperative state for this learner population, as well as the multi-systemic risks following a surgical intervention [16]. When a patient is feeling unwell in the post-operative period, there are various complications to be considered including atelectasis, pulmonary embolus, aspiration, infection, allergic reaction, or a post-operative MI. These can serve as distractors from an MI and make it difficult for the clinical clerk to make a diagnosis and initiate treatment in a timely fashion, which may be costly to the patient. 

The controlled setting of simulation-based learning achieves the balance of enabling learners to gain clinical exposure while avoiding putting patients at risk [17]. By emulating such a clinical scenario, the clinical clerks will have the opportunity to develop the competency in providing care for a patient experiencing a post-operative MI. Indeed, it is believed this novel type of learning has the potential for an increased retention of knowledge and skills compared to typical didactic learning which may improve future performance in real clinical scenarios [18].

## Conclusions

In conclusion, this clinical simulation scenario is believed to improve the time it takes to recognize and respond to a post-operative MI for clinical clerks. The ambiguity of the presenting symptoms in these patients greatly increases the difficulty in recognizing this clinical deterioration, particularly for clinical clerks who have few clinical exposures. It is often clinical clerks who are the first to check with patients in the post-operative period; therefore, it is essential they improve their ability to recognize such a red flag scenario. This clinical simulation scenario will not only benefit the medical clerks who participate, but also the clinical care team they are working with and the patient, which is the ultimate goal in any clinical setting.
